# LINC00511 is associated with the malignant status and promotes cell proliferation and motility in cervical cancer

**DOI:** 10.1042/BSR20190903

**Published:** 2019-09-13

**Authors:** Chun-Ling Yu, Xiao-Ling Xu, Fang Yuan

**Affiliations:** 1 Department of Gynaecology, Daqing Oilfield General Hospital, Daqing, Heilongjiang 163000, China; 2 Department of Gynaecology, Rizhao Central Hospital, Rizhao, Shandong 7963622, China; 3 Department of Gynecology, The Affiliated Hospital of Qingdao University, Qingdao, Shandong 266000, China

**Keywords:** biomarker, cervical cancer, LINC00511, lncRNA

## Abstract

LINC00511 is a newly identified lncRNA that is up-regulated in many types of human cancers and may serve as an oncogenic lncRNA. However, there was no report about the role of LINC00511 in cervical cancer. Therefore, we investigated the clinical value of LINC00511 in cervical cancer patients via analyzing the correlation between LINC00511 expression and clinicopathological features. Moreover, we performed loss-of-function study to estimate the effect of LINC00511 on cervical cancer cell proliferation, migration, and invasion. In our study, we found LINC00511 expression levels were increased in cervical cancer tissues and cell lines compared with adjacent normal tissues and normal cervical epithelial cell line, respectively. High LINC00511 expression was correlated with advanced clinical stage, large tumor size, histological type of adenocarcinoma, and present lymph node metastasis, distant metastasis, and poor overall survival in cervical cancer patients. The *in vitro* studies indicated that knockdown of LINC00511 inhibited cervical cancer cell proliferation, migration, and invasion. In conclusion, LINC00511 acts as oncogenic lncRNA in cervical cancer, and may be a novel biomarker and potential therapeutic target for cervical cancer patients.

## Introduction

Cervical cancer is the fourth most common female malignancy in the world accounting for approximately 6.6% of all newly diagnosed female cancers [1]. Meanwhile, cervical cancer ranks the fourth leading cause of cancer-related death in women worldwide accounting for 311365 deaths in 2018 [1]. Cervical cancer often derives from abnormal cell growth with human papillomavirus (HPV) infection on the cervix [2]. Recent decades, the generalization of disease screening and introduction of HPV vaccines jointly result in a decreasing incidence trend for cervical cancer in most countries [3,4]. Regrettably, the improvement of cervical cancer treatment strategy is limited [5,6]. Surgery, chemotherapy, and radiotherapy are still the major therapies for cervical cancer patients [7–9]. Therefore, it is necessary to investigate the molecular mechanisms of cervical cancer development for gaining novel therapeutic targets.

Long noncoding RNAs (lncRNAs) are considered a type of non-coding RNAs consisting of more than 200 nucleotides without coding protein potential [10]. More and more researches suggested lncRNAs are involved in human cancer development by regulating tumor cell proliferation, apoptosis, cell-cycle, migration, invasion, and autophagy [11,12]. Long intergenic noncoding RNA 00511 (LINC00511, also known as onco-lncRNA-12), is a newly identified carcinogenic lncRNA that maps to chromosome 17q24.3 [13]. Up to now, LINC00511 has been showed to be overexpressed in many kinds of tumor tissues, and exert oncogenic effects on lung cancer [14,15], breast cancer [16–21], pancreatic cancer [22], bladder cancer [23], osteosarcoma [24], and tongue squamous cell carcinoma [25]. In preliminary experiment, we found levels of LINC00511 expression was also increased in cervical cancer compared with adjacent normal tissues. Then, we guessed that LINC00511 functions as oncogenic lncRNA in cervical cancer. Therefore, we investigated the clinical value of LINC00511 in cervical cancer patients via analyzing the correlation between LINC00511 expression and clinicopathological features. Moreover, we performed loss-of-function study to estimate the effect of LINC00511 on cervical cancer cell proliferation, migration, and invasion.

## Materials and methods

### Clinical samples

Total 92 cervical cancer tissues and 40 adjacent normal tissues were obtained from volunteer patients with cervical cancer who underwent surgery or biopsy at Daqing Oilfield General Hospital, Rizhao Central Hospital or The Affiliated Hospital of Qingdao University. All clinical tissue samples were promptly frozen in liquid nitrogen and maintained at −80°C until RNA extraction. No radiotherapy and chemotherapy were performed before surgery or biopsy. The study procedures were reviewed and approved by the Ethics Committee of Daqing Oilfield General Hospital, Rizhao Central Hospital and The Affiliated Hospital of Qingdao University. All patients signed and provided written informed consents.

### Quantitative real time PCR

Total RNA was extracted from tissues and cells by using Trizol reagents (Invitrogen, Carlsbad, CA, U.S.A.), and transcribed into complementary DNA (cDNA) using PrimeScript RT Master Mix (Takara Biomedical Technology, Beijing, China). Then, TB Green Premix Ex Taq II (Takara Biomedical Technology, Beijing, China) was used to conducted quantitative real time PCR (qRT-PCR) at ABI 7500 PCR System (Applied Biosystems, Foster City, CA, U.S.A.) according to the instructions. The sequences of primers used in the present study were LINC00511, 5′-CGCAAGGACCCTCTGTTAGG-3′ (forward) and 5′-GAAGGCGGATCGTCTCTCAG-3′ (reverse); GAPDH, 5′-GGTGGTCTCCTCTGACTTCAACA-3′ (forward) and 5′-GTTGCTGTAGCCAAATTCGTTGT-3′ (reverse). The relative expression was standardized by GAPDH.

### Cell culture

Human cervical cancer cell lines (SiHa, HeLa, C33A, and Caski) and normal cervical epithelial cell line (Ect1/E6E7) were maintained in Roswell Park Memorial Institute (RPMI)-1640 medium with 10% fetal bovine serum (FBS) in a humidified chamber with 5% CO_2_ at 37°C.

### Cell transfection

For down-regulation of LINC00511, siRNA-LINC00511 (5′-CCCAUGUCUGCUGUGCCUUUGUACU-3′) and siRNA-NC were synthesized by Shanghai Invitrogen Biotechnology Co., Ltd. Cell transfection was performed by using Lipofectamine 3000 reagents (Invitrogen, CA, Carlsbad, CA, U.S.A.) according to manufacturer’s instructions.

### Cell Counting Kit-8 assay

Cell proliferation was estimated by Cell Counting Kit-8 (CCK-8) assay (Dojindo Molecular Technologies, Kumamoto, Japan). Transfected cervical cancer cells were (5 × 10^3^ per well) were seeded in the 96-well microtiter plates. At 24, 48, 72, and 96 h, 10 μl CCK-8 solution was added into each well, and the cells were sequentially cultured at 37°C for 2 h. The absorbance at 450 nm was detected using a microplate reader (Thermo Fisher Scientific, Waltham, MA, U.S.A.).

### Cell migration and invasion assays

For cell invasion assay, transwell chamber with Matrigel Matrix (BD Biosciences, Franklin Lakes, NJ, U.S.A.). For cell migration assay, there was no Matrigel Matrix in transwell chamber. Briefly, 600 μl RPMI-1640 medium containing with 10% FBS was added into lower chamber, and 100 μl serum-free RPMI-1640 medium with 5 × 10^4^ cells was added into the upper chamber. After 24 h culture under 37°C, cells located at upper chamber were cleaned out, and cells at lower chamber were fixed with methanol and stained with Giemsa solution. Then, a light microscope was used to count these stained cells with five randomly selected areas.

### Statistical analysis

SPSS 17.0 software (SPSS, Chicago, IL, U.S.A.) was applied for statistical analysis. All experiments were performed triplicate independently. The paired *t-*test was used for comparisons of the differential LINC00511 expression in cervical cancer tissues and adjacent normal tissues. The Student’ *t-*test was used to estimate the significance of differences between two independent groups. The relationship between the LINC00511 expression and clinical characteristics of cervical cancer patients was assessed using chi-squared test. Survival data were analyzed using the Kaplan–Meier method and Cox regression model. *P* value < 0.05 was considered statistically significant.

## Results

### LINC00511 is high-expressed in cervical cancer tissues and cell lines

**Figure 1:**
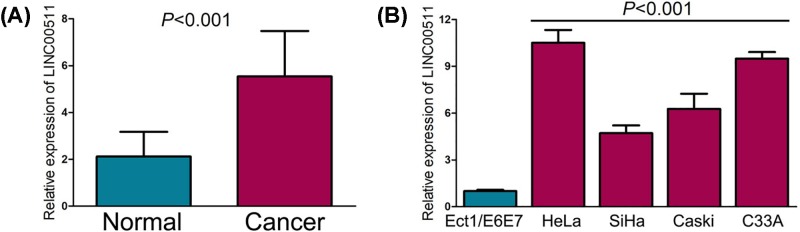
LINC00511 is high-expressed in cervical cancer tissues and cell lines (**A**) LINC00511 expression was significantly higher in cervical cancer tissues than in adjacent normal tissues. (**B**) LINC00511 expression was higher in cervical cancer cell lines (SiHa, HeLa, C33A, and Caski) than normal cervical epithelial cell line.

We first examined LINC00511 expression in 40 pairs of cervical cancer tissues and adjacent normal tissues using qRT-PCR, and found LINC00511 expression was significantly higher in cervical cancer tissues than in adjacent normal tissues (Figure 1A). Subsequently, the qRT-PCR assay also confirmed the high-expression of LINC00511 in cervical cancer cell lines (SiHa, HeLa, C33A, and Caski) as compared with normal cervical epithelial cell line (Ect1/E6E7) (Figure 1B).

### High LINC00511 expression is correlated with clinical progression in cervical cancer patients

To assess the clinical significance of LINC00511 in cervical cancer patients, we studied the correlations between LINC00511 expression and clinicopathologic parameters. All cervical cancer patients were subclassified into high LINC00511 expression group (*n*=46) and low LINC00511 expression group (*n*=46). As shown in Table 1, we observed that high LINC00511 expression was correlated with advanced clinical stage, large tumor size, histological type of adenocarcinoma, and present lymph node metastasis and distant metastasis in cervical cancer patients. However LINC00511 expression had no correction with age and HPV infection (Table 1).

**Table 1:** Relationships between LINC00511 expression and clinicopathological characteristics in cervical cancer

Characteristics	*n*	LINC00511		*p*
		High expression	Low expression	
Age (years)				
≤50	41	24	17	0.142
>50	51	22	29	
Clinical stage				
I–IIA	38	10	28	<0.001
IIB–IV	54	36	18	
Tumor size (cm)				
≤4	40	11	29	<0.001
>4	52	35	17	
Lymph node metastasis				
Absent	53	16	37	<0.001
Present	39	30	9	
Distant metastasis				
Absent	85	39	46	0.018
Present	7	7	0	
Histological type				
Adenocarcinoma	14	13	1	<0.001
Squamous cell carcinoma	78	33	45	
Histological grade				
Well	54	24	30	0.204
Moderately/poorly	38	22	16	

### High LINC00511 expression is correlated with unfavorable prognosis in cervical cancer patients

**Figure 2:**
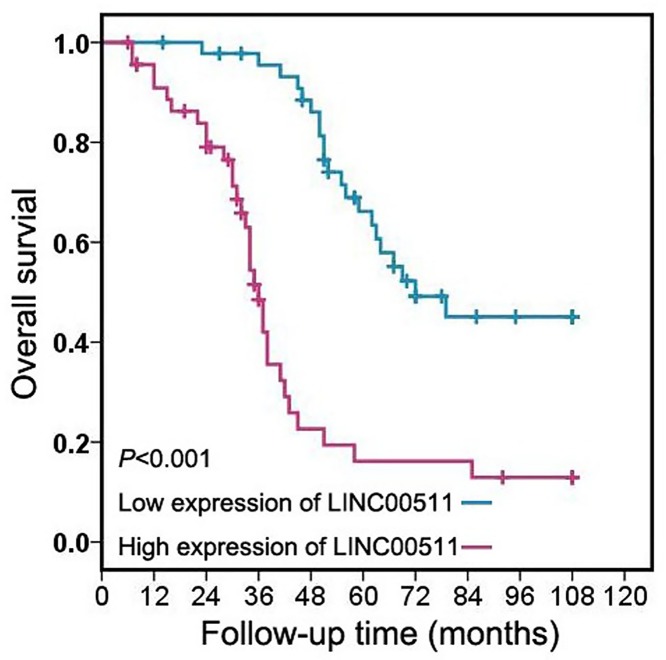
High LINC00511 expression is correlated with unfavorable prognosis in cervical cancer patients Cervical cancer patients in high LINC00511 expression group had obviously short overall survival compared with those in low LINC00511 expression group.

To further demonstrate the potential prognostic value of LINC00511, we explored the prognostic impact of LINC00511 on overall survival in cervical cancer patients. We analyzed the relationship between LINC00511 expression and overall survival of cervical cancer patients, and found patients in high LINC00511 expression group had obviously short overall survival compared with those in low LINC00511 expression group (Figure 2). Furthermore, the univariate Cox regression model indicated clinical stage, tumor size, lymph node metastasis, distant metastasis, histological type, and LINC00511 expression were identified as prognostic factors for overall survival in cervical cancer patients (Table 2). Meanwhile, high LINC00511 expression was showed to be an independent poor prognostic factor in multivariate Cox regression model (Table 2).

**Table 2:** Summary of univariate and multivariate Cox regression analysis of overall survival in cervical cancer

Parameter	Univariate analysis			Multivariate analysis		
	*P*	HR	95%CI	*P*	HR	95%CI
Age (year)						
(≤50 vs. >50)	0.429	1.252	0.717–2.185			
Clinical stage						
(I–IIA vs. IIB–IV)	0.010	2.109	1.194–3.724	0.976	1.016	0.365–2.829
Tumor size (cm)						
(≤4 vs. >4)	0.002	2.459	1.386–4.362	0.285	1.462	0.729–2.932
Lymph node metastasis						
(Absent vs. present)	<0.001	3.080	1.724–5.504	0.591	1.356	0.447–4.117
Distant metastasis						
(Absent vs. present)	0.013	3.056	1.266–7.378	0.199	0.428	0.117–1.561
Histological type						
(Adenocarcinoma vs. squamous cell carcinoma)	<0.001	0.228	0.108–0.481	0.057	0.329	0.105–1.033
Histological grade						
(Well vs. moderately/poorly)	0.872	1.046	0.605–1.810			
LINC00511 expression						
(Low vs. high)	<0.001	4.100	2.309–7.278	0.003	2.895	1.446–5.797

HR, hazard ratio; 95% CI, 95% confidence interval.

### LINC00511 acts as a tumor promoter by enhancing cell proliferation, migration, and invasion in cervical cancer

**Figure 3:**
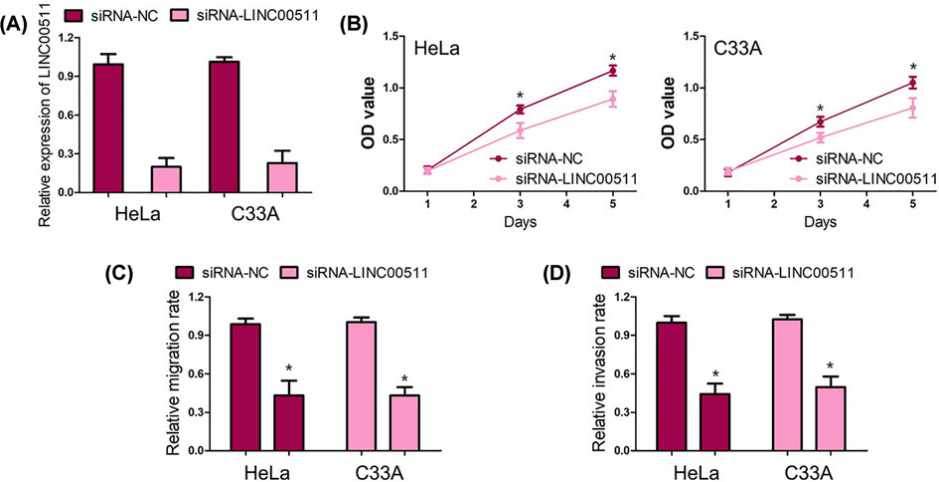
LINC00511 acts as a tumor promoter by enhancing cell proliferation, migration, and invasion in cervical cancer (**A**) HeLa and C33A cells were transfected with siRNA-LINC00511 to reduce LINC00511 expression. (**B**) Knockdown of obviously suppressed cell proliferation ability of HeLa and C33A cells. (**C**) Knockdown of LINC00511 inhibited cell migration ability of HeLa and C33A cells. (**D**) Knockdown of LINC00511 depressed cell invasion ability of HeLa and C33A cells. Each experiment was independently performed in triplicate. *P<0.001.

To evaluate the biological functions of LINC00511 during cervical cancer progression, we conducted loss-of-function study in cervical cancer cells. Then, HeLa and C33A cells were chosen for following studies *in vitro* due to relative high expression of LINC00511. First, HeLa and C33A cells were transfected with siRNA-LINC00511 to reduce LINC00511 expression (Figure 3A). The results of CCK-8 suggested knockdown of LINC00511 obviously suppressed cell proliferation ability of HeLa and C33A cells (Figure 3B). Moreover, cell migration and invasion assays revealed knockdown of LINC00511 also markedly inhibited cell migration and invasion abilities of HeLa and C33A cells (Figure 3C,D).

## Discussion

LINC00511, also known as onco-lncRNA-12, is a newly identified lncRNA that is up-regulated in many types of human cancers and may serve as an oncogenic lncRNA. Initially, Cabanski et al. conducted a pan-cancer analysis of lncRNAs comparing cancer tissue samples and matched normal tissue samples expression levels using RNA-Seq data in eight types of human cancers, and found levels of LINC00511 expression were significantly elevated in invasive breast cancer, lung adenocarcinoma, lung squamous cell carcinoma, colorectal cancer compared with corresponding normal tissues [13]. Subsequently, high expression of LINC00511 was further confirmed in lung adenocarcinoma [14], lung squamous cell carcinoma [14], breast cancer [21], pancreatic cancer [22], bladder cancer [23], osteosarcoma [24], and tongue squamous cell carcinoma [25]. However, the expression pattern of LINC00511 in cervical cancer was still unknown. Thus, we observed the LINC00511 expression in The Cancer Genome Atlas (TCGA) and The Genotype-Tissue Expression (GTEx) databases, and found LINC00511 expression was significantly overexpressed in cervical cancer tissues compared with normal tissues. Furthermore, we further confirmed the LINC00511 expression in cervical cancer tissues and cell lines through qRT-PCR, and found LINC00511 expression levels were increased in cervical cancer tissues and cell lines compared with adjacent normal tissues and normal cervical epithelial cell line, respectively. In addition, we explored the clinical significance of LINC00511 in cervical cancer patients through studying the correlations between LINC00511 expression and clinicopathologic parameters, and found high LINC00511 expression was correlated with advanced clinical stage, large tumor size, histological type of adenocarcinoma, and present lymph node metastasis and distant metastasis. Similarly, Sun et al. suggested LINC00511 overexpression was associated with large tumor size, advanced TNM stage, positive lymph node metastasis and smoking in non-small cell lung cancer patients [15]. Besides, Zhao et al. reported that high LINC00511 expression was correlated with high N stage and early recurrence in patients with pancreatic ductal adenocarcinoma [22]. In breast cancer patients, Lu et al. showed there were positive correlations between LINC00511 expression and clinicopathological parameters including TNM stages, tumor size, lymph node metastasis and distant metastases [21]. However, Ding et al. indicated LINC00511 expression had no correlation with any clinicopathological characteristics in patients with tongue squamous cell carcinoma, which may be due to small sample size [25]. Generally, LINC00511 expression was overexpressed in most types of human cancers, but more studies are necessary to explore the clinical significance of LINC00511 expression in various kinds of cancer.

The prognostic significance of LINC00511 was reported in lung cancer [15], breast cancer [18,21], and pancreatic cancer [22]. In lung cancer patients, Sun et al. found LINC00511 overexpression was correlated with short overall survival, and acted as an independent unfavorable predictor for overall survival [15]. Besides, Xu et al. and Lu et al. congruously showed breast cancer patients with high-expression of LINC00511 had poorer overall survival than patients with low-expression of LINC00511 [18,21]. Zhao et al. demonstrated that high LINC00511 expression predicted poor progression-free survival and overall survival, and served as independent prognostic indicator for overall survival of pancreatic ductal adenocarcinoma patients [22]. In our study, we also found cervical cancer patients in high LINC00511 expression group had obviously short overall survival compared with those in low LINC00511 expression group, and high LINC00511 expression was showed to be an independent poor prognostic factor for overall survival in cervical cancer patients, which was consist with the prognostic value of LINC00511 in other types of human cancer.

LINC00511 exerts oncogenic effects on cell proliferation, cell-cycle, cell apoptosis, migration, invasion, and stemness in human cancers. There was no report about the biological function of LINC00511 in cervical cancer cells. In our study, we preliminarily investigated the effect of LINC00511 on cervical cancer cell proliferation, migration and invasion. We found knockdown of LINC00511 markedly inhibited cervical cancer cell proliferation, migration and invasion. The limitation of our study is lack of the molecular mechanism of LINC00511 in cervical cancer cells. The microRNA and its target were important regulatory mechanism for LINC00511, such as miR-185-3p/E2F1 in breast cancer [21], miR29b-3p/VEGFA in pancreatic cancer [22], miR-15a-3p/Wnt signaling pathway in bladder cancer [23], miR-765/APE1 in osteosarcoma [24] and miR-765/LAMC2 in tongue cancer [25].

In conclusion, LINC00511 expression is increased in cervical cancer tissues and cell lines. High LINC00511 expression is correlated with clinical progression and poor prognosis in cervical cancer patients. Knockdown of LINC00511 inhibits cervical cancer cell proliferation, migration, and invasion.

## Abbreviations

CCK-8: Cell Counting Kit-8
HPV: human papillomavirus
lncRNA: long noncoding RNA
qRT-PCR: quantitative real time PCR
RPMI: Roswell Park Memorial Institute
